# Association of Clinical Characteristics With Variation in Emergency Physician Preferences for Patients

**DOI:** 10.1001/jamanetworkopen.2019.19607

**Published:** 2020-01-22

**Authors:** Cindy Y. Chang, Ziad Obermeyer

**Affiliations:** 1 Department of Emergency Medicine, Brigham and Women’s Hospital, Harvard Medical School, Boston, Massachusetts; 2 Berkeley School of Public Health, University of California, Berkeley

## Abstract

**Importance:**

Much of the wide variation in health care has been associated with practice variation among physicians. Physicians choosing to see patients with more (or fewer) care needs could also produce variations in care observed across physicians.

**Objective:**

To quantify emergency physician preferences by measuring nonrandom variations in patients they choose to see.

**Design, Setting, and Participants:**

This cross-sectional study used a large, detailed clinical data set from an electronic health record system of a single academic hospital. The data set included all emergency department (ED) encounters of adult patients from January 1, 2010, to May 31, 2015, as well as ED visits information. Data were analyzed from September 1, 2018, to March 31, 2019.

**Exposure:**

Patient assignment to a particular emergency physician.

**Main Outcomes and Measures:**

Variation in patient characteristics (age, sex, acuity [Emergency Severity Index score], and comorbidities) seen by emergency physicians before patient selection, adjusted for temporal factors (seasonal, weekly, and hourly variation in patient mix).

**Results:**

This study analyzed 294 915 visits to the ED seen by 62 attending physicians. Of the 294 915 patients seen, the mean (SD) age was 48.6 (19.8) years and 176 690 patients (59.9%) were women. Many patient characteristics, such as age (*F* = 2.2; *P* < .001), comorbidities (*F* = 1.7; *P* < .001), and acuity (*F* = 4.7; *P* < .001), varied statistically significantly. Compared with the lowest-quintile physicians for each respective characteristic, the highest-quintile physicians saw patients who were older (mean age, 47.9 [95% CI, 47.8-48.1] vs 49.7 [95% CI, 49.5-49.9] years, respectively; difference, +1.8 years; 95% CI, 1.5-2.0 years) and sicker (mean comorbidity score: 0.4 [95% CI, 0.3-0.5] vs 1.8 [95% CI, 1.7-1.8], respectively; difference, +1.3; 95% CI, 1.2-1.4). These differences were absent or highly attenuated during overnight shifts, when only 1 physician was on duty and there was limited room for patient selection. Compared with earlier in the shift, the same physician later in the shift saw patients who were younger (mean age, 49.7 [95% CI, 49.4-49.7] vs 44.6 [95 % CI, 44.3-44.9] years, respectively; difference, –5.1 years; 95% CI, 4.8-5.5) and less sick (mean comorbidity score: 0.7 [95% CI, 0.7-0.8] vs 1.1 [95% CI, 1.1-1.1], respectively; difference, –0.4; 95% CI, 0.4-0.4). Accounting for preference variation resulted in substantial reordering of physician ranking by care intensity, as measured by ED charges, with 48 of 62 physicians (77%) being reclassified into a different quintile and 9 of 12 physicians (75%) in the highest care intensity quintile moving into a lower quintile. A regression model demonstrated that 22% of reported ED charges were associated with physician preference.

**Conclusions and Relevance:**

This study found preference variation across physicians and within physicians during the course of a shift. These findings suggest that current efforts to reduce practice variation may not affect the variation associated with physician preferences, which reflect underlying differences in patient needs and not physician practice.

## Key Points

**Question:**

Do physicians have measurable preferences for certain kinds of patients, and is preference variation associated with overall practice variation among physicians?

**Findings:**

In this cross-sectional study of 294 915 emergency department visits, attending physicians’ preference varied significantly in how they selected the patients they saw. Accounting for these individual preferences resulted in substantial reordering of physician performance ranking by care intensity.

**Meaning:**

This study suggests that overall practice variation among physicians likely reflects preference variation, which is currently not considered in efforts to reduce practice variation.

## Teaser

This cross-sectional study examines whether variations in patient characteristics are associated with physician preferences in self-assigning or selecting patients in the emergency department.

## Introduction

Substantial variation in practice patterns across geographic areas, hospitals, and physicians (and, consequently, in health care costs) has been widely established.^1,2,3^ Considerably less evidence exists, however, on the mechanisms of this variation; specifically, why do some physicians deliver higher-intensity, higher-cost care? A common explanation for this question is that, given a similar patient (eg, one with chest pain), physicians have intrinsic preferences for higher or lower levels of care intensity (eg, whether or not to order a stress test). In the aggregate, these preferences, which are potentially driven by local practice culture, training institution, or risk tolerance, are a factor in the wide variation in practice across hospitals and regions.^4,5,6,7,8,9^

In this cross-sectional study, we explored an alternative mechanism for how variation may arise: physicians’ intrinsic preferences for different kinds of patients, who may require different levels of care intensity (eg, patients with chest pain vs toe pain). Sorting particular kinds of patients according to particular kinds of physicians is another explanation for the wide variation in care and costs across physicians (eg, patients with chest pain need different care from those with toe pain), but it has different policy implications than approaches to reduce preferences for care intensity.

To date, studies of practice variation have relied on claims data to compare regions, hospitals, or physicians. Because claims data contain limited information on physician behaviors or preferences besides care intensity and costs, they can say little about the mechanisms by which variation arises. In addition, physician preferences for patient characteristics are challenging to study in many clinical settings because physician-patient matching occurs infrequently (eg, first primary care visit), and it can be difficult to analytically isolate the role of physician preference from the role of patient preference, appointment availability, geographic barriers, insurance coverage, and other factors. The existence of preference variation may also pose methodologic problems for many practice variation studies. If patients who are sicker (or less sick) are preferentially seen by different clinicians, this practice would challenge the assumption that patients are randomly assigned to clinicians (eg, in emergency departments [EDs]).^10,11,12,13^

In this study, by contrast, we examined a setting in which physician-patient matching was frequent and easy to identify. We used a comprehensive electronic health record (EHR) data set with detailed information on physician behaviors for more than 300 000 patient encounters at a large ED of an academic hospital, including what physicians observed about patients before signing up to see them, the exact times and shift orders in which patients were seen, and detailed financial charges. In this ED, multiple physicians practiced simultaneously and independently, and physicians had the discretion to select patients. This unique setting allowed us to explore both the variation across physicians and the variation within physicians over time. Although it is important to acknowledge the main limitation of this study, that these data came from the EHR system of only 1 academic hospital, the available level of detail was, to our knowledge, not available in most data sets of this size and provides a new window into the mechanisms associated with variation.

## Methods

This cross-sectional study was approved by the institutional review board of Partners HealthCare, which also granted a waiver of informed consent given that this secondary analysis of existing data presented no more than minimal risk to participants. This study followed the Strengthening the Reporting of Observational Studies in Epidemiology (STROBE) reporting guideline.^14^

### Study Population and Setting

All adult visits to a large, urban ED of an academic hospital between January 1, 2010, and May 31, 2015, were identified. All data from adult patients and ED visits, including demographic information, previous diagnoses, attending physician of record, orders and prescriptions, and dates and times of ED arrivals and orders, were obtained from the hospital EHR system for analysis in July 2018. In this mixed-acuity ED, patients were not assigned to different clinical areas by their chief concern or acuity, and physicians were randomly assigned to these care areas in staggered, overlapping shifts.

No financial incentives were offered to physicians on the basis of patient selection or performance metrics. Physician sex and number of years after residency were obtained from the National Provider Identifier database, and physicians were categorized as having 0 to 4, 5 to 9, or 10 or more years of postresidency practice; all physicians had emergency medicine training.^15^

A total of 12 136 visits (3.9%) with incomplete time stamp, ED encounter, or attending physician information were excluded. We also excluded 15 031 visits (4.8%) seen by physicians in the bottom 25th percentile of visits per year during the study period (the median annual visits for excluded physicians were 192 visits per year).

### Observable Patient Characteristics Before Selection

The patient characteristics that can be observed in the EHR system by physicians before patient selection were age, sex, Emergency Severity Index (ESI) score, chief concerns, and comorbidities. Age, sex, ESI score, and chief concerns were recorded in triage by a nurse and were available on the ED clinical interface used by physicians to assign themselves to a patient (eFigure 1 in the Supplement). The ESI is an ED triage algorithm that stratifies patients into 5 levels from 1 (most urgent) to 5 (least urgent) on the basis of acuity and resource needs.^16^ Chief concern is the patient’s reason for the ED visit and captured as free text.

To facilitate analysis, we classified all chief concerns from ED visits using regular expression matching into 154 categories, and the highest frequency chief concerns were explored. For example, chest pain and chest discomfort were both classified into the chief concern group of chest pain. Given that comorbidities are readily accessible in the EHR system and are often reviewed by physicians before patient selection, we calculated a Gagne comorbidity score for each encounter and included it as a potentially observable characteristic.^17^ We did not examine patient characteristics that were not easily available (ie, displayed on the EHR interface) before patient selection, such as health insurance status and type or primary language spoken.

### ED Charges, In-Shift Order, and Time Control Variables

We obtained ED charges from ED administrative data. Extreme values were winsorized at the 0.05th and 99.95th percentile values (ie, values below the 0.05th percentile were replaced with the 0.05th percentile values and above the 99.95th were replaced with the 99.95th).^18^

To determine the order in which a patient was seen on a physician shift, we identified the attending physician of record and the time of first order for each visit. To account for physicians often managing multiple patients simultaneously and patients possibly not being seen in the exact order in which orders were placed, we binned patients in each given shift into up to 5 shift order groups: patients 1 to 5, 6 to 10, 11 to 15, 16 to 20, and 21 and more. For the 6453 visits (2.2%) without any orders during the ED encounter, we estimated the time of first contact from visits with similar lengths of stay (ie, by regressing the minutes to first order on length of stay for those with first order and imputing the minutes to first contact for those without an order).

Time and date of ED arrival were obtained for each visit. The month, day of the week, and hour of visit were separately recorded to account for the known temporal and demographic periodicity in ED shifts.^19,20,21^ For example, ED visit volumes are highest on Mondays and lowest between midnight and early morning, whereas presentations for influenza peak in the fall and winter and traumas peak in the summer.^22,23^ These time variables also account for the nonrandom assignment of some physicians to particular shifts, given that physicians who work only weekday daytime shifts see different types of patients than those who work predominantly on the weekends (we did not account for preference variation through the channel of shift choice). The year of visit was used to account for temporal trends in ED visits.^24,25^ Visits with time of arrival between midnight and 7 am, when all patients must be seen by the only physician on duty, were classified as overnight visits.

### Statistical Analysis

To examine the variation in observable characteristics of patients seen by different physicians, we estimated individual physician fixed effects for each patient characteristic by using multivariable regression (eg, difference in age of patients assigned to one physician over another) adjusted for a set of ED arrival time control variables. These time control variables accounted for the known temporal variation in ED shifts and nonrandom assignment of physician shifts. Primary outcomes were the variation in observable patient characteristics across different ED physicians and over the course of a shift (ie, across shift order groups). For clinical contextualization, we also stratified physicians into quintile groups by each patient characteristic and compared them across quintiles. Odds ratios (ORs) comparing physician quintiles were then obtained from the regression models of specific patient characteristics (eg, aged >50 years; ESI score of 1 or 2) on quintile groups, adjusted for time controls.

As a robustness check, we performed identical analyses for patients who presented during the overnight hours (between midnight and 7 am), when all patients must be seen by the only physician on duty in the ED. We hypothesized that, because having 1 physician overnight would leave little room for physicians to exercise choice, it would eliminate or attenuate the variation seen during the nonovernight hours (between 7 am and midnight).

To examine the variation in the observable characteristics of patients seen over the course of a shift, we similarly regressed patient characteristics on the order in which a patient was seen during a shift (ie, shift order groups 1 through 5), again adjusted for ED arrival time control variables. The ORs were calculated from regression coefficients to identify the association between in-shift order groups and patient characteristics. Data were analyzed from September 1, 2018, to March 31, 2019.

To illustrate the importance of preference variation, we quantified the extent to which preference variation accounted for the differences in the amount of care physicians delivered as measured by ED charges. First, we estimated the association between ED charges and observable patient characteristics using a regression model of ED charges on age, sex, comorbidities, ESI score, chief concerns, and time control variables. Second, we corrected the observable patient characteristics by adding the corresponding fixed effect (eg, patients seen by a physician with an age effect of +1 year will have their age adjusted down by 1 year) to simulate a scenario without preferences. Third, using multivariable regression, we estimated the charges for each encounter using the corrected patient characteristic (eg, the charges if the patient were 1 year younger), and we tabulated these corrected charges by physician. The proportion of charges attributable to preferences was determined using a regression model of the difference between reported and corrected charges on reported charges.

*F* tests were used to test for the significance of physician and fixed effects of shift order group. Two-sided *P* = .05 was considered statistically significant. Bonferroni correction was performed for multiple comparisons. All analyses were performed in R statistical software, version 3.4.3 (R Foundation). All SEs were corrected at the patient level to account for patients with multiple visits.

## Results

### Patient and ED Visit Characteristics

This study analyzed 294 915 visits to the ED seen by 62 attending physicians, representing 94.0% of all visits to the study ED during the study period after excluding encounters with incomplete patient or physician data and encounters managed by physicians with a low number of clinical encounters per year. Of the 294 915 patients seen, the mean (SD) age was 48.6 (19.8) years, and 176 690 patients (59.9%) were women. A mean of 19.0 (95% CI, 19.0-19.1) patients were seen during each shift, with 40 925 patients (13.9%) presenting during overnight hours when only 1 physician was on duty in the ED (Table 1). Many patient characteristics, such as age (*F* = 2.2; *P* < .001), comorbidities (*F* = 1.7; *P* < .001), and acuity (*F* = 4.7; *P* < .001), varied statistically significantly.

**Table 1.** Patient, Physician, and Emergency Department Visit Characteristic

Variable	No. (%)
Patient characteristics	
All patients	294 915 (100)
Female sex	176 690 (59.9)
Age, mean (SD), y	48.6 (19.8)
ESI score^a^	
Mean (SD)	2.85 (0.8)
1	4137 (1.4)
2	90 921 (30.8)
3	151 439 (51.4)
4	42 377 (14.4)
5	6041 (2.0)
Comorbidity score,^17^ mean (SD)	1.07 (2.2)
Top 10 chief concern groups	
Abdominal pain	32 186 (10.9)
Distal extremity concern	21 602 (7.3)
Chest pain	20 845 (7.1)
Dyspnea	14 756 (5.0)
Nausea, vomiting	12 952 (4.4)
Back pain	11 634 (3.9)
Headache	10 372 (3.5)
Fever, chills	9932 (3.4)
Fall	9542 (3.2)
Bleeding	9187 (3.1)
ED visit characteristics	
Time of year	
Spring (March-May)	81 774 (27.7)
Summer (June-August)	70 925 (24.0)
Fall (September-November)	67 967 (23.0)
Winter (December-February)	74 249 (25.2)
Day of week	
Weekday	215 805 (73.2)
Weekend	79 110 (26.8)
Time of presentation	
00:01-06:00	34 517 (11.7)
06:01-12:00	89 257 (30.3)
12:01-18:00	108 561 (36.8)
18:01-24:00	62 580 (21.2)
Overnight shift	40 925 (13.9)
Physician characteristics	
Female	88 666 (30.1)
Years after residency	
0-4	109 699 (37.2)
5-9	87 268 (29.6)
≥10	78 333 (26.6)

Abbreviations: ED, emergency department; ESI, Emergency Severity Index.

^a^ Scores range from 1 (most urgent) to 5 (least urgent).

### Preference Variation Across Physicians

**Figure 1.**
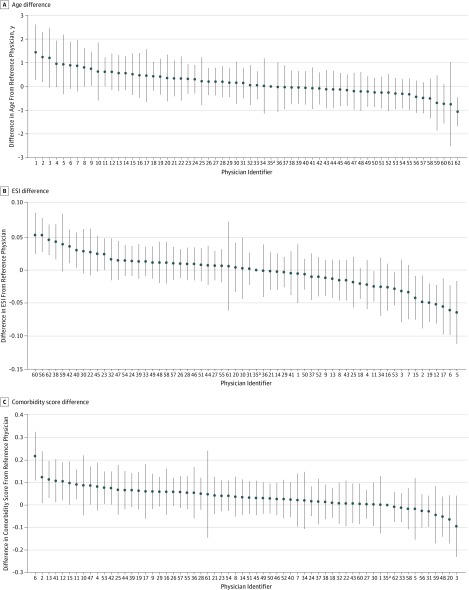
Preference Variation for Observable Patient Characteristics Across All Emergency Physicians, as Measured by Individual Physician Fixed Effects Physician identifiers were assigned according to physician rank for fixed effect for age. All differences were relative to the reference physician. The *F* score was *F*_61_ = 2.2 (*P* < .001) for the differences in age, *F*_61_ = 4.7 (*P* < .001) for Emergency Severity Index (ESI) score, and *F*_61_ = 1.7 (*P* < .001) for comorbidity score. Error bars indicate 95% CIs. ^a^Physician 35 was the reference physician.

We quantified preference variation across physicians in observable patient characteristics before patient selection. Figure 1 demonstrates that considerable variation exists in the fixed effects of patient characteristics among different physicians. Physicians in the highest quintile for patient age saw patients who were 1.8 years older (95% CI, 1.5-2.0 years) compared with physicians in the lowest quintile for age (mean age, 49.7 [95% CI, 49.5-49.9] vs 47.9 [95% CI, 47.8-48.1] years). The OR of physicians in the highest quintile for age seeing patients older than 50 years compared with physicians in the lowest quintile was 1.2 (95% CI, 1.1-1.3). The difference in mean ESI scores for the highest and lowest quintile physicians was 3.1% (2.8 vs 2.9; difference, –0.1; 95% CI, 0.1-0.1). The OR of physicians in the highest quintile for ESI score seeing patients with an ESI score of 1 or 2 compared with physicians in the lowest quintile was 1.3 (95% CI, 1.2-1.3). Physicians in the highest quintile saw patients with a 74% higher mean comorbidity score compared with physicians in the lowest quintile (1.8 [95% CI, 1.7-1.8] vs 0.4 [95% CI, 0.3-0.5]; difference, +1.3; 95% CI, 1.2-1.4). A fixed-effects model showed no statistically significant difference in the proportion of female patients seen across different physicians (*F*_1,61_ = 1.2; *P* = .15) (eFigure 2A in the Supplement). Of the top 20 chief concern groups by frequency, significant differences were found in 8 concern groups across all physicians (distal extremity concern [*F* = 2.02; *P* < .001]; chest pain [*F* = 1.33; *P* = .04]; motor vehicle–related injury [*F* = 1.34; *P* = .04]; bleeding [*F* = 1.34; *P* = .04]; ear, nose, or throat concern [*F* = 1.46; *P* = .01]; dizziness or lightheadedness [*F* = 1.41; *P* = .02]; skin concern [*F* = 1.40; *P* = .02]; and vaginal bleeding [*F* = 1.36; *P* = .03]) (eTable 1 in the Supplement).

In addition, we found associations between physician preference for age, comorbidities, and ESI score. Physicians who saw older patients also saw patients with more comorbidities (comorbidities score: 1.7 vs 0.8) and higher acuity (lower ESI score: 2.8 vs 2.9) (eFigure 3 in the Supplement). No preference variation was observed across physicians according to physician sex or years of postresidency practice (eTable 2 and eTable 3 in the Supplement).

### Preference Variation During Overnight Presentations

We hypothesized that preference variation across physicians would be attenuated during overnight hours when all patients in the ED had to be seen by the only physician on duty and there was less opportunity for patient selection. Table 2 shows a comparison of the results of *F* tests of significance for nonovernight shifts (7 am to midnight) and the overnight hours (midnight to 7 am). During nonovernight hours, statistically significant variations in age (*F*_1,61_ = 2.1; *P* < .001), ESI score (*F*_1,61_ = 4.5; *P* < .001), and comorbidities (*F*_1,61_ = 1.5; *P* = .004) were found. In contrast, with reduced discretion for patient selection during the overnight hours, no statistically significant variations were found in observable patient characteristics across physicians.

**Table 2.** *F* Tests of Significance for Preference Variation Across Physicians During Nonovernight and Overnight Hours

Variable	Shift			
	Nonovernight, Arrival Between 7 am and Midnight		Overnight, Arrival Between Midnight and 7 am^a^	
	*F* Statistic	*P* Value	*F* Statistic	*P* Value
Age	2.1	<.001	1.2	.10
Sex	1.2	.11	0.8	.90
ESI score	4.5	<.001	1.3	.07
Combined comorbidity score	1.5	.004	1.2	.14

Abbreviation: ESI, Emergency Severity Index.

^a^ When all patients must be seen by the only physician on duty.

### Physician Rankings by Care Intensity 

**Figure 2.**
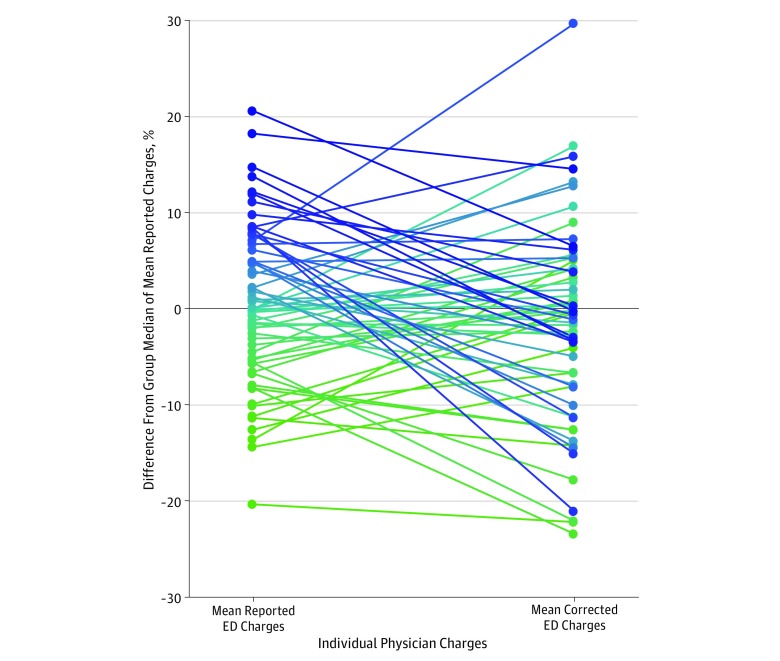
Mean Reported Emergency Department (ED) Visit Charges and Corrected ED Charges for Individual Physicians All charges were relative to the group median of the mean reported charges. Corrected charges accounted for physician preferences for observable patient characteristics (age, sex, comorbidities, Emergency Severity Index score, and chief concerns). Color gradient from green (lowest) to blue (highest) demonstrates the change in physician ranking order, with each color and data point representing a physician, ordered according to the mean reported charges.

We quantified the changes in physician rankings by care intensity, as measured by ED charges, when accounting for preference variation. Figure 2 compares the mean reported ED charges for each physician with their mean corrected ED charges, which accounted for preference. Physicians whose corrected charges were lower than their reported charges represented those with greater preference for patients with higher needs (ie, older age, higher comorbidity, and higher acuity). Accounting for physician preference resulted in considerable reordering of physician performance ranking, with 48 of 62 of physicians (77%) being reclassified into different quintiles, and in a mean absolute percentile change of 29.3 (95% CI, 24.3-34.3). Fifty-one physicians (82%) had a greater than 10th percentile absolute change in rankings, and 33 (53%) had a greater than 25th percentile absolute change in rankings. Nine of 12 physicians (75%) in the highest quintile moved into a lower quintile after accounting for preference, and 6 of 12 physicians (50%) in the lowest quintile moved into a higher quintile after accounting for preference. A regression model demonstrated that 22% of the reported ED charges were associated with physician preference.

### Preference Variation Over the Course of a Shift

**Figure 3.**
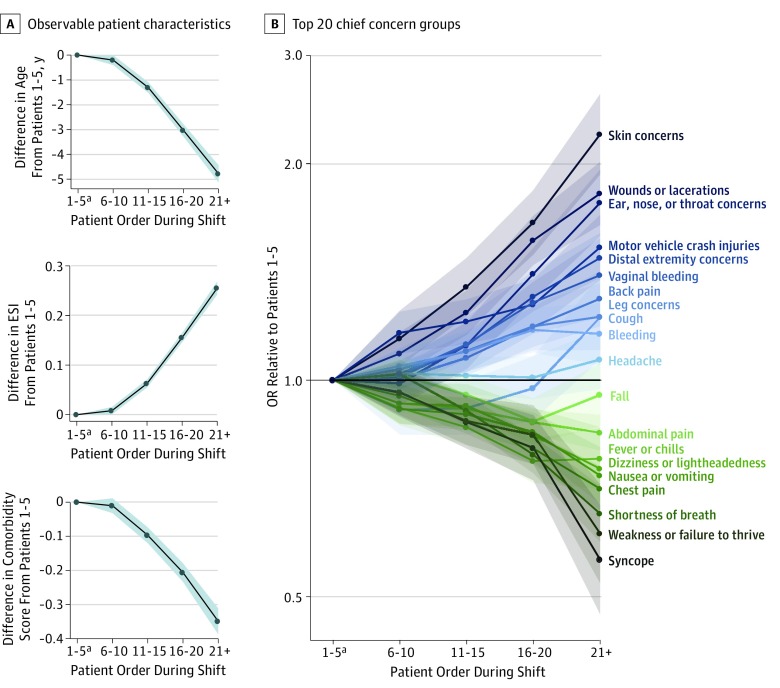
Preference Variation Over the Course of Emergency Department Shift for Observable Patient Characteristics and Top 20 Chief Concern Groups, as Measured by Shift Order Group Fixed Effects Patients in each shift were binned according to the order in which they were seen during a shift, up to 5 shift order groups: patients 1 to 5, 6 to 10, 11 to 15, 16 to 20, and 21 and more. The *F* score was *F*_4_ = 292.3 (*P* < .001) for the differences in age, *F*_4_ = 563.4 (*P* < .001) for Emergency Severity Index score (ESI), and *F*_4_ = 125.1 (*P* < .001) for comorbidity score. Shaded areas indicate 95% CIs. ^a^Patients 1-5 was the reference group.

Another unexplored possibility was that physician preference may change over the course of a shift. Figure 3A compares observable patient characteristics across the 5 shift order groups. Compared with the start of the shift, at the end of the shift, physicians tended to see patients who were younger, were female, had lower comorbidity score, and had a higher ESI score (ie, lower acuity). Patients seen at the end of the shift (group 5) were a mean of 5.1 years younger (95% CI, 4.8-5.5) than those seen at the start of the shift (group 1) (mean age, 44.6 years [95% CI, 44.3-44.9] vs 49.7 [95% CI, 49.4-49.7] years, respectively). The OR of patients older than 50 years being seen at the end of the shift compared with the start of the shift was 0.7 (95% CI, 0.6-0.7). The mean combined comorbidity score was 34% lower for patients at the end of the shift compared with the start of the shift (mean score, 0.7 [95% CI, 0.7-0.8] vs 1.1 [95% CI, 1.1-1.1]; difference, –0.4; 95% CI, 0.4-0.4). Patients at the end, compared with the start, of the shift had 7% lower acuity (mean ESI score, 2.8 [95% CI, 2.8-2.8] vs 3.0 [95% CI, 3.0-3.0]; difference, 0.2; 95% CI, 0.2-0.2). The OR of patients with an ESI score of 1 or 2 being seen at the end of the shift compared with the start of the shift was 0.5 (95% CI, 0.5-0.6). A fixed-effects model showed that physicians at the end of the shift saw 7% more female patients than at the end of shift (*F*_1,61_ = 17.4; *P* < .001) (eFigure 2B in the Supplement).

Figure 3B compares the variation in the chief concern groups over the course of the shift. At the start of the shift, physicians preferred patients with more undifferentiated chief concerns, such as syncope and weakness or failure to thrive. At the end of the shift, physicians preferred patients with more differentiated chief concerns, such as skin concerns and lacerations.

## Discussion

Using a large clinical data set from an EHR system, we observed significant variation in patient selection preference both across physicians and within physicians over the course of the shift. First, some physicians appeared to prefer patients with higher comorbidities, older age, or less defined chief concerns, whereas others preferred the opposite. Second, preferences appeared to change over the course of a shift, with younger patients, healthier patients, and patients with more defined concerns being preferred later in the shift. This variation across physicians was eliminated when analysis was restricted to overnight hours when all patients must be seen by the only physician on duty. Although physicians may not always have the option to select patients, these findings suggest that, when given the opportunity to choose, physicians likely exercise their choice based on individual preference. In addition, accounting for these individual preferences resulted in substantial reordering of physician ranking by care intensity; 75% of physicians in the highest charge quintile moved to a lower charge quintile given their preference for sicker patients. Twenty-two percent of reported ED charges were associated with physician preference.

These results could have considerable implications for how we understand and address variation in physician practice, given that varying levels of acuity or medical complexity require different clinical management.^26,27,28^ What has been labeled as *practice variation* to date (eg, different physicians manage the same patient differently) may instead reflect *preference variation* (ie, different physicians choose to see different types of patients, thereby engendering heterogeneity in practice).^13,29,30^ Physicians who prefer sicker, more complex patients would likely order more diagnostic studies compared with physicians who prefer healthier patients, and thus variation in physician practice may be associated with the differences in the types of patients the physicians prefer. These findings suggest that the commonly held assumption that patient assignments are quasi-randomized should be carefully considered when studying physician variation.

Accounting for preferences resulted in substantial reordering of physician ranking by care intensity, further illustrating the importance of recognizing the role of physician choice in evaluating performance metrics. Metrics associated with ED charges are increasingly used to compare emergency physician productivity and to determine compensation, but most metrics do not account for the types of patients seen.^31,32,33,34,35,36,37^ Case-mix characteristics are increasingly considered at the systems level, but findings from the present investigation support the need for rigorous risk adjustment when profiling physicians, even at the local level. As more programs target variation in practice across physicians, a more nuanced approach of understanding the factors associated with variation in performance measures could not only facilitate quality improvement but also mitigate physician burnout by better aligning metrics with the realities of clinical practice.

This study also found variation in preference over the course of an ED shift. Physicians chose to see patients who were younger, were healthier, had lower acuity measures, and had more defined chief concerns at the end of the shift, which demonstrated physicians’ ability to preferentially select patients. These physicians may choose younger, healthier patients at the end of shift for a variety of reasons, such as simplifying the handoff to incoming clinicians or leaving a shift on time. This proclivity, however, may also signal cognitive fatigue at the end of a shift. Given the existing literature on the association of cognitive and decision fatigue with clinical practice, further studies into the implications of time of day for patient care and outcomes may be warranted.^38,39,40^

### Limitations

This study has some limitations. It could be performed only at a single center because of the required granularity of data. The findings may not be generalizable and may apply only to EDs in which multiple physicians work simultaneously and independently and in which physicians have the discretion to select patients. The study ED has only 1 single-coverage shift per day, which limits the sample size available for investigation if preference variation decreases with reduced discretion for patient selection. We investigated the association between attending physicians and patient characteristics and not the behaviors of resident trainees or physician assistants or clinician preferences for working with certain colleagues. However, physician assistant shifts are randomly distributed across multiple care areas at several EDs, and resident trainees rotate through multiple hospital departments in monthly blocks; none of these schedules are correlated with attending physician shifts. In addition, given the large number of physician assistants and residents working in the study ED (approximately 50 physician assistants, 50 emergency medicine residents, and >150 off-service trainees), repeated pairings over multiple shifts occur infrequently. Areas of future research include the preference variation in other clinical settings and the association of preference with other physician performance metrics.

## Conclusions

Preference variation in observable patient characteristics before patient selection was found both across physicians and within physicians over the course of an ED shift. Accounting for preference variation resulted in substantial reordering of physician performance rankings. Variation in physician preference for different types of patients should be considered when evaluating physician practice variation.

## Notes

Supplement. **eFigure 1.** Emergency Department (ED) Clinical Interface That Displays Patient Age (33), Gender (Male), Emergency Severity Index (ESI: 3), and Chief Complaint (Headache) **eFigure 2.** Preference Variation for Female Patients (A) Across Physicians and (B) Across Shift Order Groups **eFigure 3.** Association Between Physician Quintiles by Age Preference and (A) Comorbidities, (B) Emergency Severity Index (ESI) **eTable 1.** Preference Variation for Top 20 Chief Complaint Groups by Frequency Across All Physicians (n = 62) **eTable 2.** Preference Variation for Observable Patient Characteristics by Physician Gender **eTable 3.** Preference Variation for Observable Patient Characteristics Across Physician Groups by Years Post-Residency (0-4, 5-9, or Over 10 Years Post-Residency)
