# Supplementary material for: Association of Clinical Characteristics With Variation in Emergency Physician Preferences for Patients
Source: JAMA Netw Open. 2020 Jan 22;3(1):e1919607. doi: 10.1001/jamanetworkopen.2019.19607 (PMC6991274; doi:10.1001/jamanetworkopen.2019.19607)
Supplement: Supplement. — eFigure 1. Emergency Department (ED) Clinical Interface That Displays Patient Age (33), Gender (Male), Emergency Severity Index (ESI: 3), and Chief Complaint (Headache) eFigure 2. Preference Variation for Female Patients (A) Across Physicians and (B) Across Shift Order Groups eFigure 3. Association Between Physician Quintiles by Age Preference and (A) Comorbidities, (B) Emergency Severity Index (ESI) eTable 1. Preference Variation for Top 20 Chief Complaint Groups by Frequency Across All Physicians (n = 62) eTable 2. Preference Variation for Observable Patient Characteristics by Physician Gender eTable 3. Preference Variation for Observable Patient Characteristics Across Physician Groups by Years Post-Residency (0-4, 5-9, or Over 10 Years Post-Residency) [file jamanetwopen-3-e1919607-s001.pdf]

Supplementary Online Content

Chang CY, Obermeyer Z. Association of clinical characteristics with variation in emergency physician preferences for patients. *JAMA Netw Open.* 2020;3(1): e1919607. doi:10.1001/jamanetworkopen.2019.19607

eFigure 1. Emergency Department (ED) Clinical Interface That Displays Patient Age (33), Gender (Male), Emergency Severity Index (ESI: 3), and Chief Complaint (Headache)

eFigure 2. Preference Variation for Female Patients (A) Across Physicians and (B) Across Shift Order Groups

eFigure 3. Association Between Physician Quintiles by Age Preference and (A) Comorbidities, (B) Emergency Severity Index (ESI)

eTable 1. Preference Variation for Top 20 Chief Complaint Groups by Frequency Across All Physicians (n = 62)

eTable 2. Preference Variation for Observable Patient Characteristics by Physician Gender

eTable 3. Preference Variation for Observable Patient Characteristics Across Physician Groups by Years Post-Residency (0-4, 5-9, or Over 10 Years Post-Residency)

This supplementary material has been provided by the authors to give readers additional information about their work.

©2020 Chang CY et al. *JAMA Network Open.*

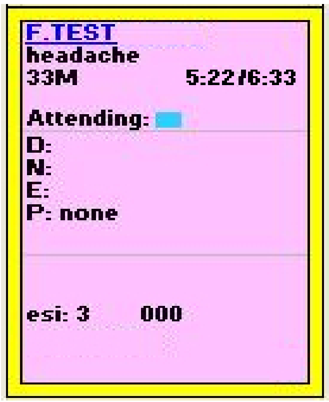

eFigure 1. Emergency Department (ED) Clinical Interface That Displays Patient Age (33), Gender (Male), Emergency Severity Index (ESI: 3), and Chief Complaint (Headache)

The interface also displays length of stay (5 hours 22 minutes since ED bed assignment, 6 hours 33 minutes since ED arrival) and care team (Attending: attending physician, D: resident physician or physician assistant, N: nurse, E: ED technician, P: primary care physician).

©2020 Chang CY et al. *JAMA Network Open.*

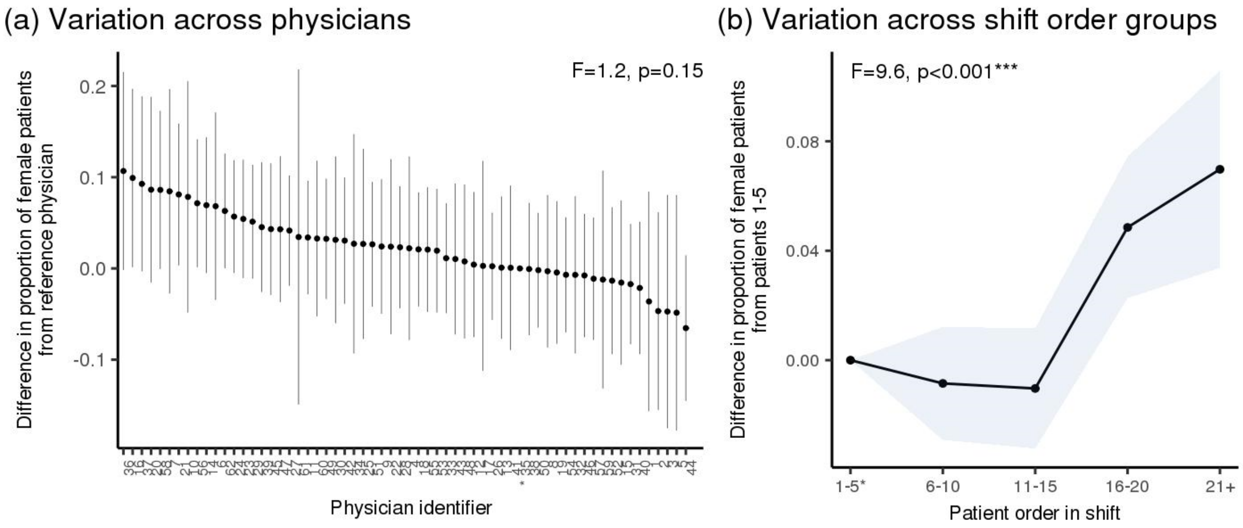

eFigure 2. Preference Variation for Female Patients (A) Across Physicians and (B) Across Shift Order Groups

Physician identifiers were assigned based on physician rank for fixed effect for age. All differences are relative to the reference physician or reference group (denoted with *).

©2020 Chang CY et al. *JAMA Network Open.*

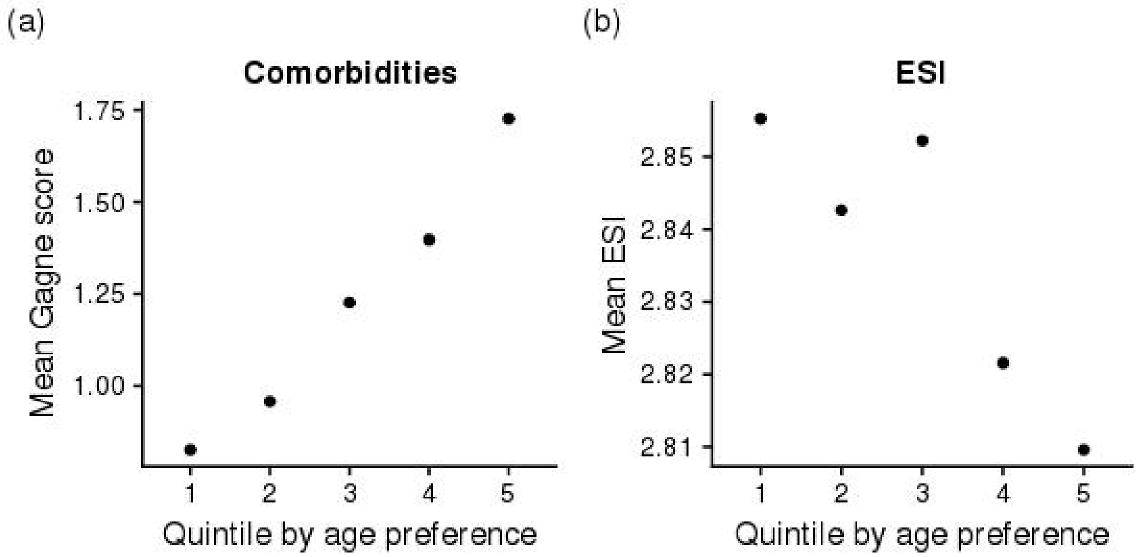

eFigure 3. Association Between Physician Quintiles by Age Preference and (A) Comorbidities, (B) Emergency Severity Index (ESI)

©2020 Chang CY et al. *JAMA Network Open.*

eTable 1. Preference Variation for Top 20 Chief Complaint Groups by Frequency Across All Physicians (n = 62)

Top 20 Chief complaint		F-statistic	F test p-value
1	Abdominal pain	1.07	0.33
2	Distal extremity complaint	2.02	<0.001
3	Chest pain	1.33	0.04
4	Shortness of breath	1.03	0.41
5	Nausea/vomiting	1.14	0.21
6	Back pain	0.83	0.83
7	Headache	1.09	0.29
8	Leg complaint	1.03	0.41
9	Wounds/lacerations	1.30	0.06
10	Fall	1.24	0.1
11	Motor vehicle injuries	1.34	0.04
12	Fever/chills	1.05	0.36
13	Bleeding	1.34	0.04
14	Ear/nose/throat complaint	1.46	0.01
15	Dizziness/Lightheadedness	1.41	0.02
16	Syncope	0.99	0.51
17	Weakness/failure to thrive	1.03	0.41
18	Rashes/lumps/bites	1.40	0.02
19	Vaginal bleeding	1.36	0.03
20	Cough	0.92	0.66

eTable 2. Preference Variation for Observable Patient Characteristics by Physician Gender

**Patient characteristics**	**F-statistic by physician gender**	**F-test p-value by physician gender**
Age	0.46	0.50
Gender	3.76	0.05
Emergency Severity Index (ESI)	0.51	0.47
Combined comorbidity score	0.22	0.64

©2020 Chang CY et al. *JAMA Network Open.*

eTable 3. Preference Variation for Observable Patient Characteristics Across Physician Groups by Years Post-Residency (0-4, 5-9, or Over 10 Years Post-Residency)

**Patient characteristics**	**F-statistic by years post-residency**	**F-test p-value by years post-residency**
Age	1.93	0.15
Gender	0.24	0.79
Emergency Severity Index (ESI)	1.3	0.27
Combined comorbidity score	0.13	0.87

©2020 Chang CY et al. *JAMA Network Open.*
